# Challenges in Medicinal and Aromatic Plants DNA Barcoding—Lessons from the Lamiaceae

**DOI:** 10.3390/plants11010137

**Published:** 2022-01-05

**Authors:** Nazia Nazar, Caroline Howard, Adrian Slater, Tiziana Sgamma

**Affiliations:** 1 Biomolecular Technology Group, Leicester School of Allied Health Science, Faculty of Health and Life Sciences, De Montfort University, Leicester LE1 9BH, UK; ads@dmu.ac.uk; 2 Tree of Life Programme, Wellcome Trust Sanger Institute, Wellcome Genome Campus, Cambridge CB10 1SA, UK; ch25@sanger.ac.uk

**Keywords:** Lamiaceae, DNA barcoding, medicinal plants, ITS region, *Ocimum*, *Lavandula*, *Mentha*, *Thymus*

## Abstract

The potential value of DNA barcoding for the identification of medicinal plants and authentication of traded plant materials has been widely recognized; however, a number of challenges remain before DNA methods are fully accepted as an essential quality control method by industry and regulatory authorities. The successes and limitations of conventional DNA barcoding are considered in relation to important members of the Lamiaceae. The mint family (Lamiaceae) contains over one thousand species recorded as having a medicinal use, with many more exploited in food and cosmetics for their aromatic properties. The family is characterized by a diversity of secondary products, most notably the essential oils (EOs) produced in external glandular structures on the aerial parts of the plant that typify well-known plants of the basil (*Ocimum*), lavender (*Lavandula*), mint (*Mentha*), thyme (*Thymus*), sage (*Salvia*) and related genera. This complex, species-rich family includes widely cultivated commercial hybrids and endangered wild-harvested traditional medicines, and examples of potential toxic adulterants within the family are explored in detail. The opportunities provided by next generation sequencing technologies to whole plastome barcoding and nuclear genome sequencing are also discussed with relevant examples.

## 1. Introduction

### 1.1. Introducing the Historical Importance and Status of Medicinal Plants

It has been well documented that herbal plants and their derivatives play critical roles in pharmaceutical, cosmetics and food industries, e.g., [1,2,3,4,5,6,7,8,9]. Historically, plants have often been selected for drug development programs because they contain specific classes of compounds, such as alkaloids and terpenoids that are known to be biologically active, or because of their traditional medicinal use [10,11,12,13]. Jumping forward in the history, these compounds have been proven to be antioxidant, antibacterial and antiviral agents with some major advantages over conventional drug therapy and limited side effects [14,15,16,17,18]. Some volatile essential oils have also exhibited a high level of antiviral activity [19,20,21]. The early 1800s was a critical point in the use of medicinal plants. In these years, the discovery and isolation of alkaloids from different plants like poppy (1806), ipecacuanha (1817), *Strychnos* (1817), quinine (1820), pomegranate (1878), and the discovery of other active substances from medicinal plants such as tannins, saponosides, etheric oils, vitamins, hormones, etc. defined the beginning of scientific pharmacy [22]. This scientific movement away from raw herbal medicines into more refined products containing only the active products created a division between what is called modern medicine and traditional medicine (TM). Nevertheless, the use of TM is still strong, and demand is actually increasing. In the recent outbreak of COVID-19, various traditional herbal plants, including members of the Lamiaceae (*Salvia* L., *Thymus* L., *Mentha* L., *Rosmarinus* L. and *Ocimum* L.), have played important roles in the treatment and recovery of individuals with COVID-19, mainly in China and India [23,24,25,26,27,28,29,30].

The WHO reported in 2014 and 2019 [31,32] that an increased number of countries are acknowledging the role of TM in their national health systems, and an increasing number of member states had developed national policies on TM, launching national laws or regulations and implementing regulations on herbal medicines [31,32]. The attention to TM from many countries is deemed to grow further. The global herbal medicines market is anticipated to reach 129 billion US dollar at the compound annual growth rate (CAGR) of 5.88% during 2010–2023 [33].

Currently in the UK, herbal medicines are regulated by the Traditional Herbal Medicines Products Directive, which was devised by the European Union. This Directive requires evidence of a plant’s traditional use as a medicine for 30 years inside the EU or 15 years in the EU and 15 years elsewhere. This has been in place since 2004; however, it came fully into effect on 30 April 2011 [34]. This means that since 2011 all manufactured herbal medicines placed on the UK market are required to have either a Traditional Herbal Registration (THR) or a Marketing Authorization (MA). It is therefore envisaged that, in the near future, all herbal medicines on the market will have to meet the same stringent criteria, satisfying EU requirements applicable to any medicine: a consistently high standard of quality, regular monitoring of safety, and full information for safe and beneficial use of the product provided by in-pack leaflets [35].

### 1.2. Increasing TM Supply Demand Threatening “Wild Type” Stock

According to recent market research reports, it is estimated that the demand for global herbal medicines will significantly increase in the future [33]. Since ancient times, a variety of products of plant origin have been used in cosmetic products, including vegetable oils, other lipids and essential oils (EOs), and are gaining popularity over synthetic products [8]. To improve the quality of food, herbs and spices have always been recognized as safe, natural preservatives to increase the shelf life of food and are excellent substitutes for chemical additives [36,37,38]. 

At least 28,187 plant species were recorded as being used medicinally [4]. Unfortunately, the increasing demand for particular herbal products has led to the scarcity of wild populations of the medicinally important species. Another factor is the non-medicinal uses of medicinal plants, including their use as natural dyes, condiments and for ornamental purposes, which is also contributing to the extremely serious threats to certain populations. This in turn increases the frequency of species adulteration—when the target plant species is, deliberately or otherwise, substituted with different species—and thereby threatens consumer safety [39]. Despite significant progress in the herbal drug industry, the quality of herbal products remains a major issue of concern [40,41,42,43,44,45], with the substitution of different species, whether intentional or unintentional, at the forefront.

### 1.3. Herbal Medicines Quality Assurance Strategies

The currently available morphologic, organoleptic and chemical detection methods such as high-performance thin layer chromatography (HPTLC), high performance liquid chromatography (HPLC), ultraviolet (UV), infrared (IR), mass spectrometry (MS) and nuclear magnetic resonance (NMR) may not be sufficient for complete plant species identification. This occurs particularly when the plant material is in a powered form and where chemical variations occur due to geographic locations and storage conditions [44,46,47]. In addition, chemical identification is not species-specific and cannot distinguish species which share chemical markers [48,49,50]. Plant identification using micro-morphological, chemical and organoleptic methods can be time-consuming, error-prone and requires expertise and reliable references [51,52,53]. In comparison, DNA barcoding is more reliable, is not affected by external factors and can be applied to all tissues [54,55]. Over the past two decades, this approach has been increasingly accepted for the identification of plants.

DNA barcoding provides a way to confirm the authentication of plants and establish a level of quality assurance within the market [52,53,54,55,56,57,58,59]. Since the first DNA barcoding study [60], the “animal barcode”, a portion of the mitochondrial gene *cytochrome oxidase* 1(*CO*1), has proved remarkably effective at discriminating among species in diverse groups, such as birds, fishes, and insects [60,61]. However, the low substitution rate of the *CO*1 gene in plants was considered unsuitable for barcoding [62]. As a result of many plant barcoding studies, e.g., [62,63,64,65,66], in 2009, the Consortium for the Barcode of Life (CBOL) Plant Working Group proposed portions of two coding regions from the plastid genome, *maturase K* (*mat*K) and *ribulose-bisphosphate carboxylase* (*rbc*L), as a standard 2-locus barcode for plants, to be supplemented with additional markers as required [67].

Proposed additional makers include the plastid intergenic spacer region of *trn*Hand *photosystem II protein D1* (*trn*H-*psb*A) and the internal transcribed spacers of nuclear ribosomal DNA (nrDNA ITS). These have generally been agreed to provide adequate resolution in a multi-locus barcode system [68,69,70,71]. Many other markers of plastid, mitochondrial and nuclear genomes, such as the *trn*L and *trn*F intergeneric spacer (*trn*L-F),RNA polymerase β subunit (*rpo*B), ATP synthase subunit b-delta (*atp*F-H), 5S-rRNA and 18S-rRNA have also been tested alone or in combination with respect to their discrimination capacity in plants and found to be appropriate for specific applications [54,72,73,74,75,76]

**Figure 1:**
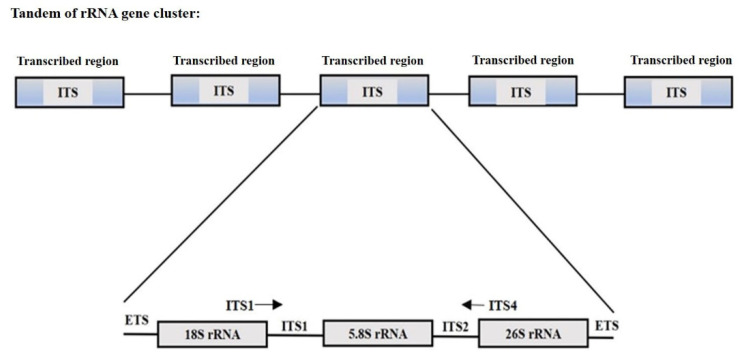
Schematic representation of nrDNA region with ITS region’s primer (ITS1/ITS4) localization (arrows). ETS (External transcribed spacers).

The nrDNA ITS is the most sequenced region across the plants with the most clearly defined barcode gap between inter- and intra-specific variations [77,78,79,80,81,82,83,84]. Components of the nrDNA ITS are ITS1, 5.8S and ITS2 regions (Figure 1) [85]. The ITS primers, ITS1 and ITS4 [86] were originally designed for fungi and found useful to detect fungal contamination in herbal plant samples [87,88,89,90].Sequences of 18S, 5.8S and 26S rDNA are highly conserved from bacteria, fungi and higher plants, enabling the design of the sequence-complemented universal primers for PCR amplification of ITS [91] across the kingdoms. To improve the quality of ITS sequence information in DNA-barcoding, there are plant-specific ITS primers that can avoid preferential amplification of fungal contaminants or non-plants templates [59,88,92,93]. Due to the decreased length of the ITS2 sequence (<300 bp), it has been proposed as a suitable for DNA barcoding applications in plants [68,94,95,96,97].There are issues, such as paralogy and polymorphic sites, with the ITS repeats [61,98] that make some taxonomists wary of using them, but for authentication purposes, ITS (and particularly ITS2) have advantages that tend to outweigh these issues. 

As is evident from the lack of consensus regarding a single universal barcode for plants, it is likely that a more flexible approach will be required in order to make the best use of this technology for the benefit of consumers [99]. The British Pharmacopoeia, when introducing DNA barcoding for plant drugs, recognized this and approached each medicinal plant as a new ‘target species’ inhabiting a particular taxonomic environment. This enabled molecular markers to be selected for each target species, after analysis of each of the standard barcode regions, providing both identification of the target species and differentiation from potential adulterants [58,99]. The BP chose the *trnA-psbA* region of *Ocimumtenuiflorum* L. (Holy Basil Leaf) as their first exemplar of DNA barcoding as a tool for botanical identification, and the selection process is described in detail by Sgamma et al. [58] (see Example 1 of the Supporting Information in their publication).

The strategic application of DNA-based identification methods is best applied with a careful consideration of the specific trade, economic and taxonomic environment inhabited by a medicinal plant. The human preference for plant varieties or species based on characteristics that are desired for particular industries exerts a strong selective pressure and skews the material available on the international market toward the leading demand. This presents a challenging situation for those wishing to trade medicinal plants, as this is often not the primary market demand, and the material available may or may not represent the original, traditional, medicinal profile (genetic or phytochemical). These issues are described in this review using various Lamiaceae species as case studies to show the pressures of different markets and how these in turn effect the beneficial application of DNA-based authentication methods.

## 2. DNA Barcoding—Lessons from the Lamiaceae

The Lamiaceae (mint) is one of the largest families of aromatic plants and contains about 237 genera and 7756 species [4,100]. Of these, 1056 species are used as medicinal plants which is about 13.7% of the family, representing a higher-than-normal proportion [4]. The widely known herbal genera of the family such as *Lavandula* (lavender), *Mentha* (mint), *Ocimum* (basil), *Scutellaria* (skullcap), *Thymus* (thyme) have significant medicinal properties and are also major commodities in the food industry [100,101,102]. The Lamiaceae family has great diversity and variety with cosmopolitan distribution and inhabits different natural ecosystems. Some species of the family (e.g., lavender, basil, mint, oregano and thyme) are cultivated due to the high demand for medicines and food from individual species [101]. Many species are known for their *aromatic* properties due to the production of EOs in the *glandular trichomes*, one of the significant features of the family [103,104,105,106]. The plants in the family produce an enormous variety of compounds that act as attractants and defence molecules in nature and are also widely used by humans [107,108,109,110]. The EOs typical of the family are rich in terpenoids such as monoterpenes, iridoids and sesquiterpenes which are responsible for many of these functions. 

Therefore, herbs and spices from this family are important in the pharmaceutical, flavouring, perfumery and cosmetic industries [111,112,113,114,115,116]. Global supply chains and consumer demand for particular characteristics exert selective pressure, and result in discrete and specific identification and authentication scenarios when attempting to select medicinally relevant material. These issues can be well described using case studies within the complex and species-rich Lamiaceae. *Mentha* (Mint) exemplifies a scenario of extremely strong consumer demand based around a particular EO, carvone. This skews the global market towards high yield varieties and is further complicated by ready hybridisation and human intervention via widespread cultivation and has led to traders adulterating their products to fulfil market demands. *Lavandula* (Lavender) is a vital contributor of fragrance industry and most famous for its relaxing aromatic qualities. Increasing demand for lavender extracts in the current market situation is due to two strong economic drivers, scent and horticulture. This dual pressure has resulted in a two-tier trade with varieties selected preferentially for one or the other driver. The rising demand for lavender products and the higher prices charged for English lavender due to its lower oil production per plant have led to lavender adulteration in the market. *Ocimum* (Basil) is widely used in systems of indigenous medicine and food. Migration of cultures from south Asia to different parts of the world has resulted in basil species becoming intermingled, making DNA authentication assays more difficult to interpret [117]. *Origanum* (Oregano) as a spice is utilised in numerous regions of the world. The herb has a strong culinary consumer demand and is widely cultivated for this purpose. Oregano is the name used to refer to a great variety of plants. Sixty-one species from seventeen genera in six different families are known as oregano [118], exemplifying the problem of trying to match scientific species with common plant names. Along with the existence of synonymous names, the adulteration of herbs may also be economically motivated and intentional. *Scutellaria* (skullcap) is mainly used in the pharmaceutical industry and misidentification due to high morphological similarities with its adulterants can lead to serious health issues [119]. *Salvia* (sage) is the largest genus of the Lamiaceae; most of the plants of this genus are well known for their nutritional components.

Thus, accurate plant identification is essential, in order to reduce the potential risks to the consumers’ well-being and safety. The benefit of DNA-based authentication in these arenas is considered, and how the objectives and approach of work must flex to fit the particular issues faced is discussed. 

### 2.1. A Carvone Focussed Market and Hybridisation: Mentha L.—Mentheae: Nepetioideae 

**Figure 2:**
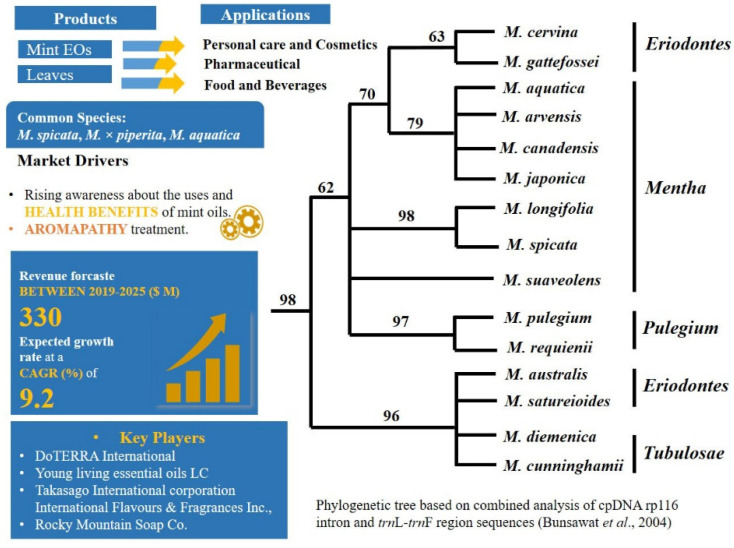
Mintproducts market analysis [130] and phylogenetic relationship among the species.

The genus *Mentha* (mint) is an important aromatic plant and consists of 24 species and 15 hybrids [120] and it is in high demand because of its carvone EO content (Figure 2). Some of the common species of *Mentha* such as *M. aquatica* L. (watermint), *M. arvensis* L. (cornmint), *M. longifolia* L., *M.* × *piperita* L. (peppermint), *M. pulegium* L., *M. × rotundifolia* (L.) Huds. and *M. spicata* L. (spearmint) are commonly grown for the production of EOs and/or utilized as food flavouring and medicinal agents in many countries of Europe, Australia, America, and the Middle East [121,122,123,124].

Based on cpDNA data, the genus is strongly supported as monophyletic (Figure 2), however, a phylogenetic understanding within the *Mentha* has always been challenging and it may be attributed to a high incidence of polyploidy, variation in base chromosome number, diverse morphology, vegetative propagation, and frequent interspecific hybridization both in wild and cultivated population [125,126,127,128,129]. The basic chromosome number of the genus is x = 12, but complex hybridization processes have led to a large diversity of chromosome numbers from diploid to octoploid [127].

*M. spicata* (2n = 48) has long been thought to originate by hybridization between *M*. *longifolia* and *M*. *suaveolens*, with a doubling of the chromosome number. However, in a recent study no evidence was found that *M*. *spicata* is of hybrid origin and revealed that many cryptic species were underestimated in subsection *Spicatae* [131]. Spearmint is widely grown throughout all regions of the world and the leaves possess a characteristic aromatic odour and pungent taste. Carvone is the main constituent of spearmint oil [132].

*M*. × *piperita* is a hybrid between *M*. *spicata* and *M*. *aquatica* [133]. The aromatic compounds of the genus, such as menthol, menthofuran, carvone, linalool, and linalyl acetate are frequently used as a part of confectionary, as flavor enhancing agents in toothpastes, chewing gums and beverages, bakery, cosmetics, as oral hygiene products, pharmaceuticals and pesticides [134,135]. Leaves, flowers and stems have been used as herbal teas and spices in many foods to add aroma and flavor [136,137]. The content of aromatic compound differs between species and also depends upon seasonal variations, soil types, etc. [138,139]. Peppermint has a characteristic aromatic odour and taste, with a cooling sensation on the breath, and menthol (35–40%) is the primary constituent of peppermint oil [140,141,142]

Carvone is a very important monoterpene ketone and occurs at high concentrations (70–80%) in spearmint oil and is also the major component responsible for its aroma [143]. Carvone can be used to identify spearmint, but it is also abundant in other species such as caraway (*Carum carvi* L.) and dill (*Anethum graveolens* L.), which consequently present major adulteration issues [144]. Another example is peppermint, with a large quantity of global demand produced in US. Peppermint EO has great importance in the flavour and food industries because of its unique sensory and quality properties. Cornmint, a less expensive mint plant is grown in India and is frequently used as a peppermint adulterant [49,129,145]. Another adulterant of peppermint is spearmint EO and L-menthol, which could be identified by using attenuated total reflectance-Fourier transform infrared (ATR-FTIR) spectroscopy coupled with partial least squares regression (PLSR) and principal component regression models, described in a recent study by Taylan et al. [146]. The DNA sequences *rbc*L, ITS, *mat*K, *trn*H-*psb*A, *atp*B and *atp*C have been used as an approach to distinguish and identify the complex relationships among *Mentha* species [129,147,148,149,150]. The whole plastid genomes of *M*. *spicata* (Accession no.NC_037247.1), *M*. *longifolia* (Accession no. NC_032054.1) and *M*. *× piperita* (Accession no. NC_047475.1) have been sequenced and characterised to develop conservation strategies, metabolic engineering, molecular breeding and accurate identification of taxa [151,152]. Due to morphological, genetic plasticity and variation in active components of Eos with respect to geographic origin of *Mentha* species/subspecies/cultivar, accurate identification is essential for explanation of phylogenetic relatedness and distinctive marker profiles at the DNA level. 

There are basically two types of challenges in the correct identification of *Mentha* species:

(i) Hybridization or cryptic taxa. Hybridization and polyploidy have indeed most likely played important roles during speciation in mints, which forms one reason the number of taxonomically valid species is a subject of controversy [153,154].The complex genomic networks of taxa with porous genomes, cause phenotypic mosaics that behave dynamically [155]. Indeed, plasticity is highly known in *Mentha* [156,157], which confounds morphological identification. Complex morphological, chemical, and molecular diversity in mints have already been described in many studies, e.g., [126,131,158,159,160,161,162,163,164,165,166]. Despite the enormous amount of data gathered, however, there is still need of taxonomic revisions within the genus. In the recent revised phylogenetic analysis [131] the origin of *M*. *spicata* as hybrid was not supported and hidden cryptic taxa were detected in the genus.
(ii) Selection of chemical markers. Carvone, a characteristic compound produced by *M*. *spicata* is also produced by different species from different plant families [144]. Chemical markers such as carvone in spearmint, and menthofuran and menthol in peppermint are used in practice for authentication of oils regardless of their sources [49].

Therefore, there is need to design a combination of approaches in case of mint, where the misidentification or presence of hidden cryptic species hybridisation makes DNA methods difficult, and production of characteristic compounds in other species makes chemical analysis problematic. Furthermore, in the case of the molecular approach, attempting to use a single, universal DNA barcoding region in these cases would be unproductive, as it would ignore levels of genetic divergence associated with different reproductive strategies. It would be more productive for a DNA authentication approach to target multiple plastid DNA markers to overcome these problems. Obviously, intraspecific plastid DNA polymorphism is highly possible and maternal transmission of the chloroplast species of hybrid origin would not be differentiable from the maternal parental species. Therefore, a multi-level barcoding strategy should be used, testing for both nrITS and multiple plastid markers to increase resolution. Another important aspect, often forgotten in DNA barcoding experiments, is the number of samples analysed. It has been previously suggested that barcoding analysis should use a minimum of 10 individuals per species [167], which could overcome possible ambiguous results.

### 2.2. Two-Tier Trade Variety Selection for the Fragrance and Horticulture Industries: Lavandula L.—Ocimeae: Nepetioideae

EOs are used frequently in both the flavour/food and fragrance industries and the demand is steadily expanding. The market value of EOs worldwide is expected to grow from around 17 billion U.S. dollars in 2017 to about 27 billion U.S. dollars by 2022 [168]. United Kingdom export of essential oils, perfumes, cosmetics, toiletries was 5.33 billion U.S. dollars during 2020 [169]. The demand for EOs is increasing each year and is expected to grow further in the next few years. The main drivers are growing consumer awareness and a rising demand for high quality natural components in personal care products and in perfumes. 

A large quantity of EOs is utilized by the fragrance or flavour industries, with only a small percentage for therapeutic purposes. In order to lower the price of the EOs, adulterants are added to the oils by some producers. It is estimated that approximately 80% of commercially available EOs are adulterated in some way [170]. Major adulterants of EOs are vegetable carrier oils, cheaper oils of the same species but of different geographical origins, EOs extracted from another part of the plant, cheaper EOs from related species, and synthetic compounds [171]. Low quality EOs are prone to produce allergic reactions, irritations, and/or toxic side effects, particularly to young and old populations who are more susceptible [172,173].

**Figure 3:**
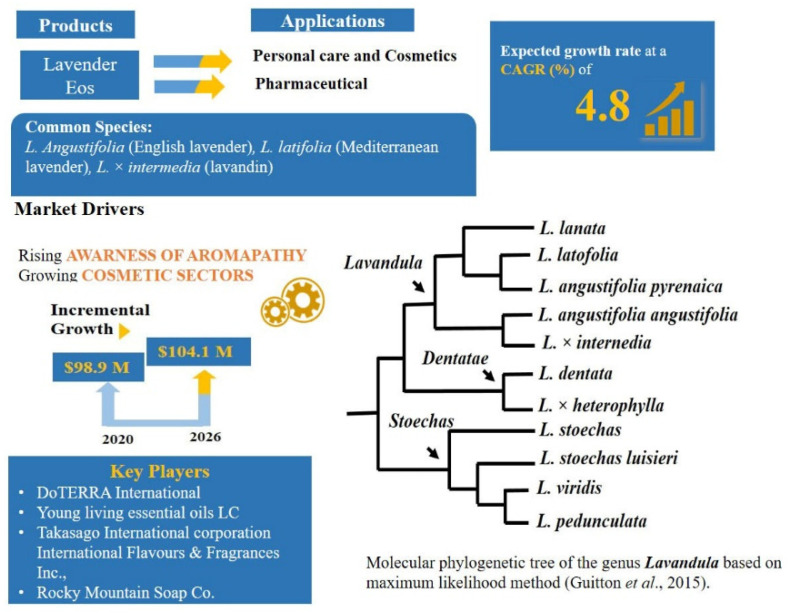
Lavender products market analysis [180] and phylogenetic relationship among the species.

The *Lavandula* (Lavender) are aromatic flowering plants that include 41 species and are widely distributed across Europe, northern and eastern Africa, the Mediterranean, south-west Asia, Arabia, western Iran and India [174,175,176]. Bulgaria is the world largest producer of lavender oil nowadays. However, France and China are among those countries that have also increased their lavender production [177]. The results of phylogenetic studies [178,179] based on cpDNA *trn*K-*mat*K partial sequences confirmed the monophyly of *Lavandula* (Figure 3) and the section classification of the genus as defined by Alan [175].

The lavender EOs are applied in a wide range of home and personal care products, perfumery, aromatherapy and alternative medicine [181]. Lavender EOs have a long history of use as fragrance and aromatherapy ingredients. The plant is used in traditional and folk medicines in different parts of the world for the treatment of several gastrointestinal, nervous and rheumatic disorders and is also used for anxiety, stress and insomnia [182,183,184]. Over the years, application of lavender extract, oil and essence in food and beverage products has also increased to a substantial level and is forecasted to grow at an increasing rate in each sector (Figure 3).

Lavender is classified into four categories: *L. angustifolia* Mill. (English Lavender), L. stoechas L. (French Lavender), L. latifolia Medik. (Mediterranean lavender) and *L.* × *intermedia* (lavandin, which is a cross between L. latifolia and *L. angustifolia)* [185]. Lavender oil, obtained from the flowers of L. angustifoliais chiefly composed of linalyl acetate (3,7-dimethyl-1,6-octadien-3yl acetate), linalool (3,7-dimethylocta-1,6-dien-3-ol), lavandulol, 1,8-cineole, lavandulyl acetate, and camphor [186,187]. English lavender oil is considered to have unique properties that are beneficial for the skin, hence it is used in various skincare products. It is a general view that English lavender is mainly grown for the perfume industry, but they are also grown as scented ornamental plants because of their aroma and attractive blue flowers. The oil from the English lavender plant attracts a high value and is often adulterated with EOs from the much cheaper sterile hybrid, lavandin (*L.* × *intermedia*) that produces more oil per plant [188]. Another factor contributing to the adulteration of English lavender with lavandin could be linked to climate change, as lavender production is affected by the weather, with an impact on availability and price [188]. The price is also influenced by the origin of cultivation of the plant, with French grown plants considered to have the oil with the best quality and, therefore, the highest prices [177]. The less valuable lavandin oil is graded accordingly to the origin of production and the hybrid used [188].

Adulteration of lavender can occur in different ways. The Lavender oils could be adulterated with similar oils from different *Lavandula* species or hybrids, or by the addition of synthetic components with a similar chemical composition, or with non-volatile solvents [189]. Using chemical tests, it is possible to differentiate between Lavandin and lavender oil [188]. 

In many cases, therefore, intentional adulteration is driven by economic reasons. On the other hand, accidental contamination may occur due to the high level of hybrids. Although lavender oils can be tested and differentiated by chemical fingerprint tests, this is not always reliable as many factors, including environment and developmental stage could alter the oil composition. Therefore, these tests could give us an indication of the oil quality but not always link this to the oil origin. Companies that want to check the quality of their starting material could benefit from DNA barcoding as a faster and more reliable way of testing the authenticity of *Lavandula* plants before assessing the quality of the lavender oil.

Traditionally, morphological features such as the size and shape of leaves, the presence or absence of non-glandular or glandular trichome and inflorescence were used to distinguish distant lavender species from one another [175]. A number of DNA barcoding studies have been done so far in the case of *Lavandula*. Hindet al. [190] tested molecular markers such as *matK*, *rbcL*, *trn*H-*psb*A and ITS to identify important lavender species. The plastid markers *rbcL* and *trn*H-*psb*A alone did not discriminate between *L*. *angustifolia*, *L*. *latifolia* and *L*. x *intermedia*. The ITS concatenated with *rbcL*, *trn*H-*psb*A and *rbcL*+*trn*H-*psb*A were able to discriminate the cultivated *L. latifolia* from *L. angustifolia* and *L. × intermedia*. The *matK* barcode was not amplified in this study as also reported in previous studies specifically for Lamiaceae taxa [64,191]. In another study the *matK* gene was successfully applied to differentiate nine *Lavandula* species along with high-resolution melting (HRM) analysis [192].

### 2.3. The Diaspora of People and Plants: Ocimum L.—Ocimeae: Nepetioideae

The tremendous increase in migrations and diasporas of human groups in the last century not only bring challenging issues for societies, but also create dramatic changes in traditional knowledge, beliefs and practices related to medicinal use of plants [193]. The discrepancy between traditional and scientific nomenclature often goes unnoticed, and these discrepancies become highly problematic for quality control and consumer protection in the importing countries.

**Figure 4:**
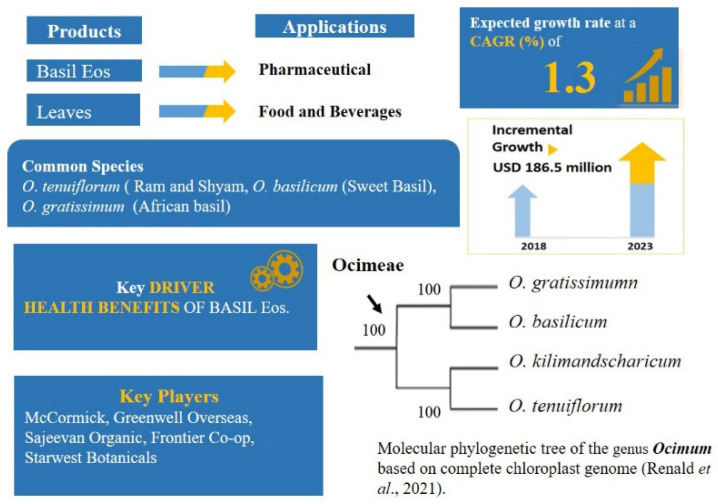
Basil products market analysis [199] and phylogenetic relationship among the species.

*Ocimum* is one of the best-known genera of the family for its medicinal properties and economically important aromatic oils (Figure 4). This genus is monophyletic [175], highly variable and possesses a wide range of intra- and inter-specific genetic diversity, comprising more than 65 species distributed all over the world [194,195,196]. *Ocimum* species and varieties have unique and individual chemical compositions, but their medicinal properties have not been fully explored.Moreover, due to extensive and nonregulated collections, many species have become threatened or endangered [197,198].

Among these, *O. tenuiflorum* L. (Holy basil or Tulsi), is an important medicinal plant, with religious significance to the Hindu community throughout the world and worshipped for over more than 3000 years due to its healing properties [200,201,202,203]. Tulsi plants are characterised by having a complex chemical composition, containing many biologically active phytochemicals with variable proportions among varieties. The EOs of tulsi contain phenylpropanoids such as eugenol, methyl eugenol, chavicol and estragole (methyl chavicol) [204]. Two chemotypes of *O*. *tenuiflorum* are known as ‘*Ram*’ (white) and ‘*Shyam* or *Krishna*’ (black) have been identified based on high or low methyl eugenol:eugenol ratios [200]. As methyl eugenol and methyl chavicol are classed as genotoxic carcinogens, it is important to ensure that the levels of these compounds in herbal products fall below the regulatory thresholds. The genus is known to possess antibacterial, antianaphylactic, antihistaminic, wound healing, antidiabetic, larvicidal, anti-genotoxic, neuro-protective, cardio-protective, hepato-protective, anti-carcinogenic and mast cell stabilization activity [205,206]. *O. basilicum* L. (Sweet Basil), *O. gratissimum* L. (African basil or Vana tulsi as some authors claim) and O. *tenuiflorum*, are frequently cultivated in several countries of East Asia, Europe, America, and Australia for the production of EOs [207,208,209].

An important aspect of globalization of plants is the migration of seeds/plants, and of the traditional knowledge of indigenous medicinal plants along with the migration of people. Tulsi seeds/plants were brought to UK from Africa and India. It was later revealed in a DNA barcoding study [117] that during this migration “Rama tulsi” used by south Asian communities in UK had been substituted with African *O*. *gratissimum*. Out of four barcoding markers (*matK*, *rbcL*, *trn*L-F and *trn*H-*psb*A) tested by Jurges et al. [117], *trn*H-*psb*A was identified as the best marker for commercial application to discriminate different types of Tulsi—“Rama Tulsi” and “Krishna Tulsi” of *O*. *tenuiflorum* and “Vana Tulsi” of *O. gratissimum*. These plastid markers also clarified the phylogenetic relationships mirrored in the chemical differences within the *Ocimum* [117]. Rama and Krishna appeared within the main clade of *O*. *tenuiflorum* and Vana within a different clade as observed in previous studies [210,211]. The *trn*H-*psb*A region was introduced as the most suitable candidate barcode into the British Pharmacopoeia [212] to authenticate *O*. *tenuiflorum* in industrial quality assurance procedures. 

Another approach was adopted by Ríos-Rodríguez et al. [213], who designed a trait-related DNA barcode based on the enzyme eugenol *O*-methyltransferase (EOMT), responsible for the synthesis of methyl eugenol. The study revealed that a multiplex PCR coupled with trait-related and trait-independent markers can differentiate *O. tenuiflorum* from other *Ocimum* species and identify methyl eugenol chemotypes of *O. tenuiflorum*, even in dried material sold as mixtures, confirming the results of Mali [200]. The high degree of intra- and inter- specific genetic diversity in the genus determines a large number of subspecies, different varieties and forms which produce EOs with varying chemical composition [214]. Some of the *Ocimum* species are highly similar in apparent vegetative morphology and are hence misidentified. Moreover, the cultural and commercial values associated with the Tulsi plant have also increased the risk of adulteration [215]. Different species are sold mostly as dried powders under the same name, and therefore there exists a great need to develop an accurate method that can prove the authenticity of plant raw material. The existing methods to ensure correct plant species collection and cultivation include organoleptic traits and phytochemical methods [216,217,218], but none of these methods sufficient to guarantee the authenticity of the plant [219]. 

### 2.4. Demands of High-Quality Herbal Products in the Food Market: Origanum L.—Mentheae: Nepetioideae

**Figure 5:**
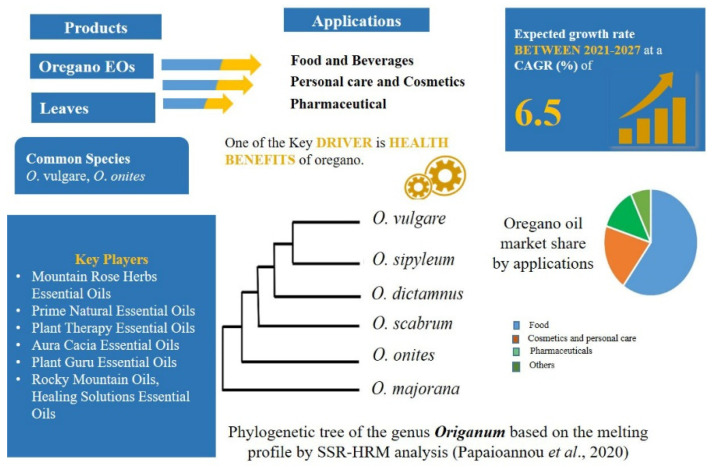
Oregano products market analysis [221] and phylogenetic relationship among the species.

The demand for spices and herbs is increasing globally, and this trend is anticipated to continue in the coming years (Figure 5). The expected growth is forecast to be driven by increasing interest in international ethnic cuisines combined with heathy eating trends. Due to increased awareness and demand, food safety issues such as adulteration of herbs and spices has been recorded frequently, as mentioned previously. Some of the most widely used culinary herbs, such as basil, thyme, mint and oregano are from Lamiaceae. These herbs have been used since ancient times to improve the characteristic of food, as natural preservatives and for their nutritional properties [145,220].

The *Origanum* genus is comprised of up to 43 species and 16 hybrids characterized by a high morphological and chemical diversity [222,223]. They are all confined to the Mediterranean region except for *O*. *vulgare*, which has a native geographical range which extends from Macronesia throughout Europe and eastward to China [224].

The *Origanum* species have been used since ancient times as culinary and medicinal herbs. Medicinally, *O. vulgare* (oregano) has been used for thousands of years as a stimulant, carminative, expectorant, and tonic to cure asthma, cough, indigestion, rheumatism, toothache and insect bites and as preservatives in meat storage [225,226,227]. Oregano EO is composed of different compounds. The majority is thymol and carvacrol, but other compounds include p-cymene, thymoquinone, and γ-terpinene [228,229,230]

Oregano is often commercialized as a fine powder or a mixture of small fragments of dried leaves, which makes morphological recognition difficult. Several herbs including oregano leaves/oils are used both in both the food and pharmaceutical industries and the usage is anticipated to rise by a considerable rate (Figure 5). There are many species of the genus used around the world as “oregano”, but variations in their bioactive compounds have been reported in different studies [231,232,233,234]. Geographical distribution and harvest season also effect the chemical composition of the oregano plants [235].

Oregano is the name used to refer to a great variety of plants based on its particular aroma, with at least sixty-one species and seventeen genera belonging to six different families known as oregano [118]. Oregano EOs and spices are frequently adulterated with different genera/species from the same family (e.g., *Saturejamontana* L. and *O*. *majorana* L.) and from different families (e.g., *Rubus* spp., *Cistus ×incanus* (Rosaceae), *Rhus coriaria* (Anacardiaceae), *Pimpinella anisum* (Apiaceae), *Myrtus* spp. (Myrtaceae), *Corylus avellana* L. (Betulaceae), *Olea europaea* L. (Oleaceae) and *Triticum aestivum* L. (Poaceae) [236,237,238,239]. The quality of oregano spices is standardised by using protocols based on those specified by European Pharmacopoeia, and only these two species, *O*. *vulgare* and *O*. *onites* L., can be commercialized as true oregano [239,240]. Within the food market, criteria approved by American Trade Association and ESA for spices are limited to the phytochemical profile of EOs, weight by weight, and the acid-insoluble ash contents. These are time-consuming and not particularly discriminative in the case of oregano, where contamination may be perpetrated with misidentified or cheaper spices belonging to the same genus. 

DNA barcoding approaches have been the most effective tools currently used for the authentication of herbal products, particularly when coupled with HRM analysis—a novel analytical approach. The United States Food and Drug Administration (FDA) supports the use of DNA-based technologies in quality assurance of herbal products, among other innovative analytical technologies [241]. In the case of oregano, a universal sequence of the *trn*L-intron barcode from different *Origanum* species was identified [226]. When the molecular marker was coupled with HRM analysis, it was found to be an effective method to discriminate *Origanum* species and genotypes in a fast and simple way [242].

### 2.5. Rising Demand of Natural Products in Pharma Market: Scutellaria L.—Scutellarioideae

*Scutellaria* is an herb, commonly known as skullcap, which contains approximately 478 species [243] and has a cosmopolitan distribution [100,244]. Several species have a long history of being used as traditional herbal medicines to treat respiratory, neurological and cardiovascular diseases, hepatic and gastric disorders [245,246,247]. The flavonoids and many other active chemicals derived from *S*. *baicalensis* (Huang Qin), *S*. *barbata* and *S*. *lateriflora* have been found to possess anticancer characteristics [50,79,248,249,250]. Due to the outstanding medicinal value, the chemical composition of the genus has attracted considerable attention in the past ten years. A wide range of chemical components have been discovered from the genus, however, the flavonoids and diterpenes are the two main groups of active constituents in this genus [251].

**Figure 6:**
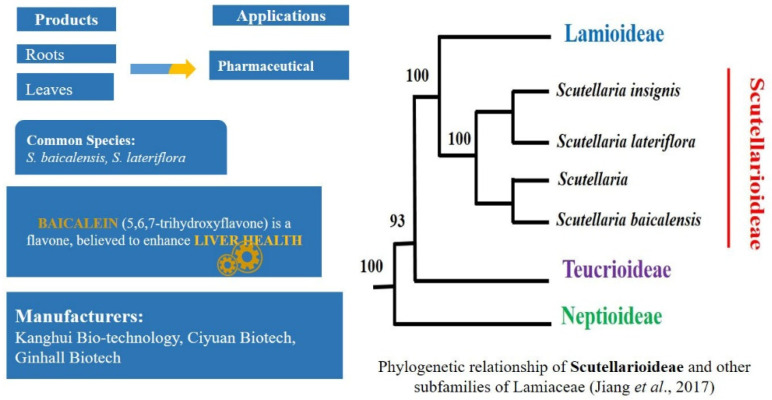
Skullcap products, applications and phylogenetic relationship among the species of Scutellarioideae and other subfamilies of Lamiaceae.

The main flavonoids are baicalin, baicalein, wogonoside and wogonin, which possess wide pharmacological activities and are produced in high concentration in different parts of different species (Figure 6) [252,253,254,255,256].The flavonoids in the roots of *S*. *baicalensis* were found to be high compared to the aerial parts whereas in *S. lateriflora* the flavonoid content of the aerial part, especially the leaf, was more than in the root [79,257].

**Figure 7:**
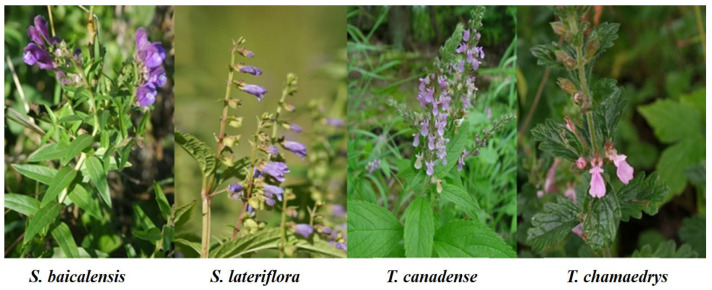
Morphological similarities between *Scutellaria* and *Teucrium*.

The intentional or unintentional adulteration of *S. lateriflora* herbal products with hepatotoxic *Teucrium spp*. (Germander), *T*. *canadense* and *T. chamaedrys*, as well as different species from the same genus *Scutellaria* has been reported since the early 1990s [119,258,259,260]. The genus *Teucrium* also belongs to the same family Lamiaceae and has high morphological similarities with *Scutellaria* (Figure 7). Despite these morphological similarities, in the most recent classification of Lamiaceae based on molecular phylogeny, the genera *Scutellaria* and *Teucrim* have been placed in different subfamilies; Scrutellarioideae and Ajugoideae, respectively [261]. Phylogenetic analysis based on chloroplast genome sequences suggested that Scutellarioideae is a sister taxon to Lamioideae (Figure 6) [262]. 

A variety of successful analytical methods for the quality control of skullcap raw material and products were applied to measure the chemical differences between *Scutellaria* and *Teucrium*. The genus *Scutellaria* contained flavonoids, while the major phenolic components of the two *Teucrium* species (*T*. *canadense* and *T*. *chamaedrys*) were the phenylethanoids, verbascoside and teucrioside. The phenylethanoid marker was suggested to distinguish between the two genera [245,259,263,264,265,266]. However, these methods require expert analysts and are time consuming.

DNA barcoding has also been tested for authentication of the species. Three candidate DNA barcodes *mat*K, *rbc*L and the *psb*A-*trn*H were sequenced and analysed by Guoet al. [267] to discriminate *S*. *baicalensis* and its adulterants (*S*. *amoena*, *S*. *rehderiana*, and *S*. *viscidula*) and this study proposed multilocus barcodes *rbc*L+ *psb*A-*trn*H for the detection of species authentication. We have designed HRM primers (a “two set strategy”) to target SNPs of *rbcL* and *trnH*-*psbA*, that are able to differentiate *S. lateriflora* from other species of the same genus, and from *Teucrium* spp. (unpublished data). Our preliminary results also confirmed that *rbcL* is best suited for discriminating plant taxa at the genus level, while *trnH*-*psbA* is a suitable candidate for design of species-specific barcoding tests, confirming the results of Guo et al. [267].

### 2.6. High Utilisation of Functional or Superfood Food and Complex Taxonomy: Salvia L.—Mentheae: Nepetioideae 

The genus Salvia, with about 980 species is the largest genus in the angiosperm family Lamiaceae. It is distributed throughout the subtropical and temperate regions of the Old World and the New World [268,269,270,271,272]. Many species of the genus have been widely utilised in the pharmaceutical, food, cosmetic and horticulture industries [272,273]. The genushas health-healing properties such as antiseptic, antipyretic, analgesic, antimicrobial, antioxidant, anticancer, anticholinesterase and anti-inflammatory characteristics [274]. Different parts of the *Salvia* plant such as leaves, flowers, roots and seeds may be used for their health benefits and have played an important role in the treatment and recovery of individuals with COVID-19 [275].

**Figure 8:**
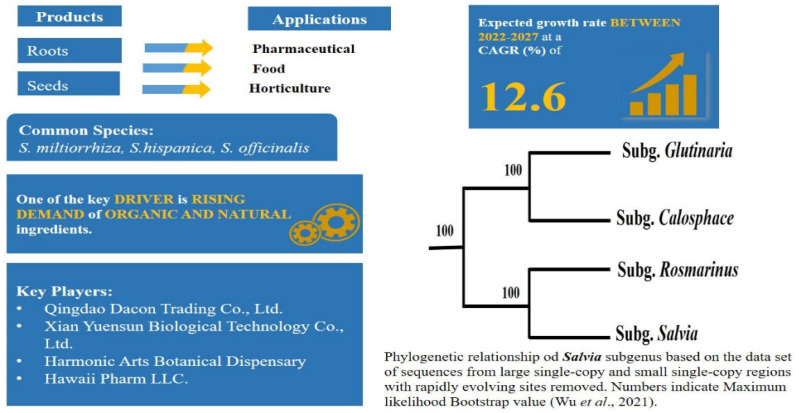
*Salvia* products, applications and phylogenetic relationship among subgenera.

*S. miltiorrhiza* (‘Danshen’ in Chinese) is used in traditional Chinese medicines to treat cardiovascular and cerebrovascular diseases and hyperlipidaemia [272,276]. *S. hispanica*, commonly known as “Chia”, was initially cultivated by Mesopotamian cultures as staple food and medicinal plant in pre-Columbian times [277]. It was rediscovered in the middle of the 20th century and is now available commercially worldwide as a superfood [278]. Chia seeds contain healthy omega-fatty acids and other nutritional components [272,279,280]. *S. divinorum* has been used in religious rites by Mazatec shamans to induce hallucinatory visions [281]. In addition, around 150 species are used in the horticulture trade, such as *S*. *officinalis* (common sage), *S*. *elegans* (pineapple sage), *Salvia splendens* (scarlet sage) and others (Figure 8) [282].

The genus is well-known for its unusual diverse staminal morphology, in which two fertile stamens are separated by a significantly elongated connective tissue, which form a lever mechanism important in pollination [283]. Based on floral or morphological characters different classification schemes within the genus were proposed, e.g., [284,285,286,287,288,289,290]. On the basis of molecular phylogenetic studies, traditionally defined *Salvia* is non-monophyletic and is classified into 11 subgenera [268,270,271,272,291]. However, to understand the inter and intra-specific relationships of the genus, it has been suggested in a recent plastomic study that using large single copy and small single copy regions with the exclusion of more rapidly evolving sites could produce the highest resolution in the phylogenetic analysis of *Salvia* (Figure 8) [292].

Like other species of the Lamiaceae, species of Salvia are under constant threat of economically motivated adulteration. For instance: (i) the roots of *S. miltiorrhizaare* adulterated with roots of *S. przewalskii*, *S. yunnanensis*, and *S. trijug,* (ii) sage leaves are adulterated witholive leaves, myrtle leaves, sumac, hazelnut leaves, *Cistus* and *Phlomis*, strawberry tree leaves and sandalwood [293], (iii) chia oil is expensive to produce and can therefore be easily adulterated with cheaper oils such as corn oil, peanut, soybean and sunflower [294]. Analytical techniques, such as gas chromatography mass spectroscopy (GC-MS) and FTIR, have been used to detect adulterants in *Salvia* species [293,295], however these techniques require expertise and can be time consuming as described earlier.

Wang et al. [273] conducted a comprehensive DNA barcoding study by using different DNA markers: *rbc*L, *mat*K, *trn*L-F, *psb*A-*trn*H and ITS1 alone or in different combination for the identification purpose of *Salvia* species. In this study, ITS1 was found to be superior when compared to other markers for discriminating between species, especially *S. miltiorrhiza*. In a recent study, DNA barcoding was coupled with chemical analysis by LC-MS profiling and this dual approach proved to be a powerful tool in identification of taxonomically close *Salvia* species [296]. High-throughput sequencing of chloroplast genomes has also been successfully used for discrimination of species within the genus [275]. Multiple approaches have been tested so far for the authentication of economically important species in *Salvia*; however, there is still a need to develop quick and simple identification techniques. DNA barcoding can also be used to address conservation issues and germplasm preservation. Identification of plant species is a fundamental component of conservation and management planning, and the benefits of molecular identification include that it can be done any time of the year and from very small tissue samples [297,298]. In the case of *Salvia*, despite its importance all over the world, a significant number of the species, for example, *S.* *pentstemonoides* (Big red sage), *S. taraxacifolia* and *S. miltiorrhiza* (red sage) are listed as threatened or endangered [299,300,301]. Particular attention is needed to design conservation strategies for their protection. 

## 3. Evolving DNA Barcoding Technologies

The conventional method of generating DNA barcodes for a species or a specimen are through PCR amplification and Sanger sequencing methods. However, Sanger sequencing technology has been found to be inadequate in some respects when compared to next-generation sequencing (NGS) technology [302,303,304]. The NGS techniques are increasingly used in many fields to obtain huge amounts of data and discover novel and essential information about the genomes. In terms of plant DNA barcoding, different approaches such as transcriptome analysis, whole chloroplast genome sequencing and mini barcoding have been developed by using NGS techniques. 

Transcriptome sequence data from plants greatly increases the opportunities for identification of additional loci as DNA barcodes and measuring the phylogenetic relationships among various taxa. Rastogi et al. [196] reported the comprehensive transcriptome analysis of *Ocimum* species and identified transcriptome SNPs and SSR markers that could be used for the identification of closely related taxa in the genus. Likewise, SNP data was discovered from transcriptome assemblies of *Lavandula* clones to differentiate between *L. angustifolia* and its hybrid *L. latifolia* [305].

The strategy of using the whole chloroplast genome to identify species and reconstructing phylogenetic relationships between closely related species has also been successfully applied to Lamiaceae species. In *Mentha*, *Ocimum*, *Lavandula*, *Origanum* and *Scutellaria*, chloroplast genome sequencing is being carried out to understand the complex relationships between species and genera, the function of genes and the medicinal nature of the metabolites synthesized in the plant [152,196,262,306,307,308,309,310,311,312]. Access to the whole chloroplast genome will also provide more informative barcoding sites and has the potential to improve the plant identification process between closely related species. However, the genetic information in angiosperm chloroplasts is mostly inherited maternally, making the chloroplast genome a good indicator only of maternal ancestry [313]. To identify hybrids (e.g., *Mentha*), the use of chloroplast genome sequences alone are not sufficient and can be concatenated with markers from nuclear genomes to establish a standardised barcoding system in these species.

DNA mini-barcoding, using a smaller length of DNA, 100–250 bp in length with sufficient variable sites could be a solution to overcome the difficulties associated with traditional DNA barcoding [50,313,314,315]. Based on specially designed primers, mini-barcodes can accurately identify targeted species within a genus or family [50]. Moreover, in cases where samples contained different contaminations, identification methods combined with NGS can identify species from multiple taxa [316,317,318,319]. 

Species adulteration or contamination can cause severe adverse effect on human health, as reported in the cases of *Origanum* and *Scutellaria*. The quality control of the plant material is critical and its enforcement seems to be necessary for the protection of the consumer. In addition, global and competing marketplaces added to the decline of the natural habitat of traditional medicinal plants, threaten herbals with extinction. Work to understand the mechanisms of traditional medicines is therefore urgent and must be based on the ‘wild type’ material to conserve the link with thousands of years of traditional knowledge.We know that this is useful based on the number of pharmaceuticals developed from medicinal plants and we risk squandering the collective knowledge.This work is only achievable using a combination of authentication methods.

## Data Availability

The data presented in this study are openly available in [repository name e.g., FigShare] at [doi], reference number [reference number].
